# Highly Cancer Selective Antiproliferative Activity of Natural Prenylated Flavonoids

**DOI:** 10.3390/molecules23112922

**Published:** 2018-11-09

**Authors:** Agnieszka Bartmańska, Tomasz Tronina, Jarosław Popłoński, Magdalena Milczarek, Beata Filip-Psurska, Joanna Wietrzyk

**Affiliations:** 1 Department of Chemistry, Wrocław University of Environmental and Life Sciences, C.K. Norwida 25, 50-375 Wrocław, Poland; tomasz.tronina@upwr.edu.pl (T.T.); jaroslaw.poplonski@upwr.edu.pl (J.P.); 2 Department of Experimental Oncology, Hirszfeld Institute of Immunology and Experimental Therapy, Polish Academy of Sciences, Weigla 12, 53-114 Wrocław, Poland; milczarek@iitd.pan.wroc.pl (M.M.); filip@iitd.pan.wroc.pl (B.F.-P.); wietrzyk@iitd.pan.wroc.pl (J.W.)

**Keywords:** antiproliferative activity, hop flavonoids, selectivity, phytoestrogen

## Abstract

Xanthohumol (XN) and four minor hops prenylflavonoids: α,β-dihydroxanthohumol (2HXN), isoxanthohumol (IXN), 8-prenylnaringenin (8PN), and 6-prenylnaringenin (6PN), were tested for antiproliferative activity towards human cancer and normal cell lines. Nonprenylated naringenin (NG) was used as a model compound. Xanthohumol, α,β-dihydroxanthohumol and 6-prenylnaringenin were the most active compounds. Xanthohumol exhibited higher antiproliferative activity than cisplatin (CP) against five cancer cell lines: ovarian resistant to cisplatin A2780cis, breast MDA-MB-231 and T-47D, prostate PC-3, and colon HT-29. Isoxanthohumol was more potent than cisplatin against breast cancer cell lines MDA-MB-231 and T-47D whereas 6-prenylnaringenin was stronger than cisplatin against breast cancer cell line T-47D. It was found that tested chalcones possessed highly selective antiproliferative activity towards all tested breast cancer lines compared to the normal breast MCF 10A cell line (the calculated selectivity index ranged from 5 to 10). Low antiproliferative activity of naringenin indicates the importance of the prenyl group with respect to antiproliferative activity.

## 1. Introduction

Hop cones (hops) are a rich source of prenylated flavonoids. The major flavonoid found in hops with 0.1–1% of dry weight is xanthohumol (XN), the chalcone with a very broad spectrum of biological activities, such as antioxidant, anti-inflammatory and anticancer ones, suggesting a potential chemo-preventive effect [1,2,3].

Other prenylated flavonoids occurred at 10–100 fold lower concentrations than xanthohumol and, therefore, they were studied less intensely with the exception of 8-prenylnaringenin (8PN)—the most potent phytoestrogen so far known [4].

Although xanthohumol is the major flavonoid found in hops, during wort boiling it undergoes thermal isomerisation to isoxanthohumol (IXN), which becomes the major hop flavonoid in beer (500 µg/L–3.44 mg/L) [5] and therefore also in the human diet. 8-Prenylnaringenin is produced in small amounts in a non-enzymatic way during drying, storage and extraction of hops, and also in an enzymatic way along with 6-prenylnaringenin (6PN) from desmethylxanhtohumol during wort boiling. Moreover, isoxanthohumol is the precursor of 8PN, which can be converted to 8PN by demethylation, catalysed by the cytochrome P-450 enzymes [6] or intestinal microbiota [7]. The above information indicate that the effect of 8-prenylnaringenin on the human organism is not directly dependent on the 8PN dose. Hop flavonoids have chemopreventive properties, i.e., they suppress carcinogenesis and tumor development, and also therapeutic activity, because they exert cytostatic effect and induce cancer cell apoptosis. However, their influence on the risk of estrogen-dependent cancer development is not unequivocal. Phytoestrogens may be implicated in the etiology of breast cancer, and are being evaluated as potential cancer chemopreventive or promoting agents [8,9,10].

So as to evaluate the effects of xanthohumol, isoxanthohumol and 8-prenylnaringenin on breast cancer tissue, Bolca and co-workers assessed their concentration, nature of metabolites, and biodistribution and compared them to 17β-estradiol [11]. Phase I metabolism appeared to be minor, whereas extensive glucuronidation was observed (>90%). Total flavonoids in breast adipose/glandular tissue and their derived estrogen equivalents were negligible compared with 17β-estradiol. Probably low doses of tested compounds are unlikely to elicit estrogenic responses in breast tissue.

The most common treatment for breast cancer patients involves using doxorubicin and docetaxel. However, therapeutic effects of these anticancer drugs are frequently accompanied by harmful side effects and also chemoresistance of cancer cells is often observed. Hence, there is a constant search for new effective and safe drugs [12]. A part of this study is the work of Liu and co-workers who demonstrated that the MCF-7/ADR cell line, which is resistant to doxorubicin, was sensitive to xanthohumol. It was found that the combination of XN and the commonly used anticancer drug doxorubicin showed a strong synergistic effect on MCF-7/ADR cells. Therefore, MCF-7/ADR cell line may be treated either with xanthohumol alone, or in combination with other anticancer drugs [13].

Delmulle and co-workers studied the antitumor activity of prenylated flavonoids against two prostate cancer cell lines: PC-3 and DU 145. In the case of PC-3 cells treated with isoxanthohumol and 6-prenylnaringenin and DU 145 cells treated with IXN, 8PN and 6PN, the formation of vacuoles was observed. The results showed that IXN, 6PN and 8PN, but not XN, induced a caspase-independent form of cell death and seem to be promising drugs for cancer therapy [14].

De Vincenzo and co-workers studied the relationship between chalcone structures and their cytotoxic activity against ovarian cancer cells (OVCA433). They compared a chalcone to the analogous α,β-dihydrochalcone and drew the conclusion that saturation of the α-β double bond decreases the chalcone activity [15].

Brunelli and co-workers demonstrated that 8-prenylnaringenin inhibits estrogen receptor-alpha-mediated cell growth and induces apoptosis in MCF7 cells [16].

Wang and co-workers studied the effect of flavonoids on MCF 10A and MCF7 breast cancer cell lines. They proved that 6-prenylnaringenin preferentially affected the mechanism of detoxification of estrogens in both the cell lines. 8-Prenylnaringenin enhanced the metabolism of MCF7 breast cancer cells only to a small degree, whereas isoxanthohumol and xanthohumol had no effect at all on the metabolism of tested cell lines. The results suggest that 6-prenylnaringenin has anticancer properties, however, further research in this area is needed [17]. Recently, much attention has been focused on cancer-preventive properties of prenylflavonoids. It was found that chrysin derivatives, prenylated or geranylated at C-6 or C-8 positions, are effective inhibitors of multidrug resistance proteins, which are responsible to a large extent for the failure of anticancer chemotherapy [18,19].

Fukai and co-workers investigated a range of low molecular weight polyphenols against oral tumor cell lines. They showed higher cytotoxicity to squamous cell carcinoma (HSC-2) and human salivary gland tumor (HSG) cell lines than to normal human gingival fibroblasts HGF. Moreover, the presence of a hydrophobic group (prenyl or geranyl) did not considerably change the cytotoxic activity of chalcones, flavanones and non-flavonoid compounds, whereas prenylation of isoflavones and 2-arylbenzofuran significantly enhanced their cytotoxic activity [20].

The results obtained by Henderson and co-workers suggest that the hop flavonoids are potent and selective inhibitors of human cytochrome P450. Thus, the in vitro effects of these phytochemicals on cDNA-expressed human CYP1A1, CYP1B1, CYP1A2, CYP3A4 and CYP2E1 were examined by the use of diagnostic substrates and the carcinogen AFB.

The prenylated chalcone, xanthohumol (XN), almost completely inhibited (EROD) the activity of CYP1A1 and CYP1B1. At the same concentration, other hop flavonoids showed varying degrees of inhibitory action ranging from 99.3 to 1.8%. The most effective inhibitors of CYP1A2 acetanilide 4-hydroxylase were 8-prenylnaringenin (8PN) and isoxanthohumol (IX) [21].

Inhibition studies showed that the xanthohumol, 8-prenylnaringenin and isoxanthohumol strongly inhibited the mutagenic activation of IQ mediated by cDNA-expressed human CYP1A2 in the Ames Salmonella assay. The three prenylflavonoids also markedly inhibited the human CYP1A2-mediated binding of IQ to metabolites that bind to DNA. The inhibition of the metabolic activation of IQ was paralleled by the inhibition of acetanilide 4-hydroxylase activity of human CYP1A2 [22].

Thus, xanthohumol, isoxanthohumol, and 8-prenylnaringenin are potent inhibitors of the metabolic activation of procarcinogen and may have the potential to act as chemopreventive agents against cancer induced by carcinogens (e.g., heterocyclic amines) activated by CYP1.

If the flavonoids and other polyphenols present in the diet are CYP1 substrates, then inhibition of these enzymes appears to be disadvantageous, because most studies on the possible mechanism of the cancer-protective effect of dietary flavonoids, hydroxylated by CYP1, have assumed that free hydroxyl groups of flavonoids and other polyphenols are necessary for their biological effects (as an antioxidants). However, in the human body, dietary polyphenols are rapidly transformed by glucuronosyltransferases and sulfotransferases. Thus, methoxylated compounds are more metabolically stable, increasing their bioavailablity. Recently, it was shown that tumour-specific enzymes can catalyze the *O*-demethylation of methoxylated flavones, resulting in the formation of flavones with free hydroxyl groups. It was proposed that demethylation of methoxylated flavones is another example of bioactivation of naturally occurring prodrugs [23].

There are prodrugs that have little or no significant cytotoxic effect in their normal state, but which are highly cytotoxic after hydroxylation by CYP1B1. Then, a self-targeting drug delivery system should be provided in which the patient is administered a non-cytotoxic compound (or low cytotoxic), e.g., in a systemic way, and then hydroxylated in tumor cells to produce a highly cytotoxic compound that acts to kill cell’s cancer. The fact that CYP1B1 is not expressed by normal cells means that the hydroxylation of the compound occurs only at the site of the tumor cells and, therefore, only the cancer cells are affected, thus providing a self-feeding drug delivery system [24].

Naringenin (NG), the flavonoid present in human diet (though contained in hops in a very small amounts) is an antioxidant and hence may exert a cancer-protective effect. On the other hand, naringenin is hydroxylated by human CYP1 enzymes at the 3′ position to give eriodictyol, which may be involved in the induction of carcinogenesis in mammals. However, nowadays, CYP1 substrates, in contrast to CYP1 inhibitors, are considered as anticancer drugs [23,24]. Flavones contained in the diet, such as naringenin, at low concentrations, inhibit aromatase activity (CYP19) and therefore inhibit estrogen biosynthesis, exerting an antiestrogenic effect, which is important in breast and prostate cancers treatment [25,26].

Stompor et al. studied cellular accumulation and cytotoxicity of naringenin and 8PN in normal (BJ) and cancer cells (U-118 MG). Obtained data indicated that 8PN exhibited higher cytotoxicity against the used cell line than NG and the effect of both flavones was stronger in U-118MG cells than in normal fibroblasts. Moreover, naringenin demonstrated higher selectivity for cancer than normal cells [27].

## 2. Results and Discussion

### 2.1. Chemistry

Xanthohumol was isolated by extraction from spent hops (variety “Marynka” crop 2017) with ethyl acetate with a yield of 0.38% according to the procedure described by Tronina et al. [28]. Isoxanthohumol was obtained by chemical conversion of xanthohumol with a yield of 62.3%. α,β-Dihydroxanthohumol (2HXN) was obtained by chemical synthesis with 85.0% yield. Demethylation of isoxanthohumol to 8-prenylnaringenin was achieved with 75.0% yield, and prenylation of naringenin to 6-prenylnaringenin with 5.2% yield. Naringenin was purchased from Sigma-Aldrich. The purity of all tested compounds was over 98%.

### 2.2. Antiproliferative Activity In Vitro

Cancers in each stage involve uncontrolled cell division. There are reports stating that polyphenols inhibited cellular transformation and proliferation in several studies in vitro [18,29]. We decided to examine hop flavonoids towards human cancer cell lines, mainly those arising from female sex hormone imbalance.

In our earlier study on the antiproliferative activity of xanthohumol and its biotransformation products, we demonstrated that the aglycone was more active than its sugar derivatives [29] and xanthohumol H (3′-[3′-hydroxy-3”methylbutyl]-4,2′,4′-trihydroxy-6′-methoxychalcone) (the last compound was inactive towards PC-3 cell line) [30]. These observations were the inspiration for the present research.

**Figure 1:**
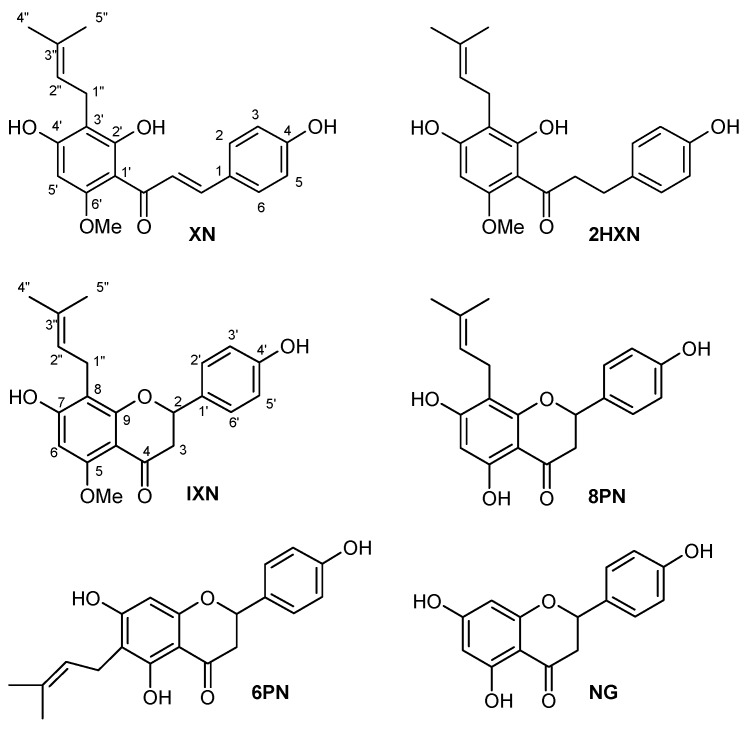
Chemical structures of tested flavonoids.

**Figure 2:**
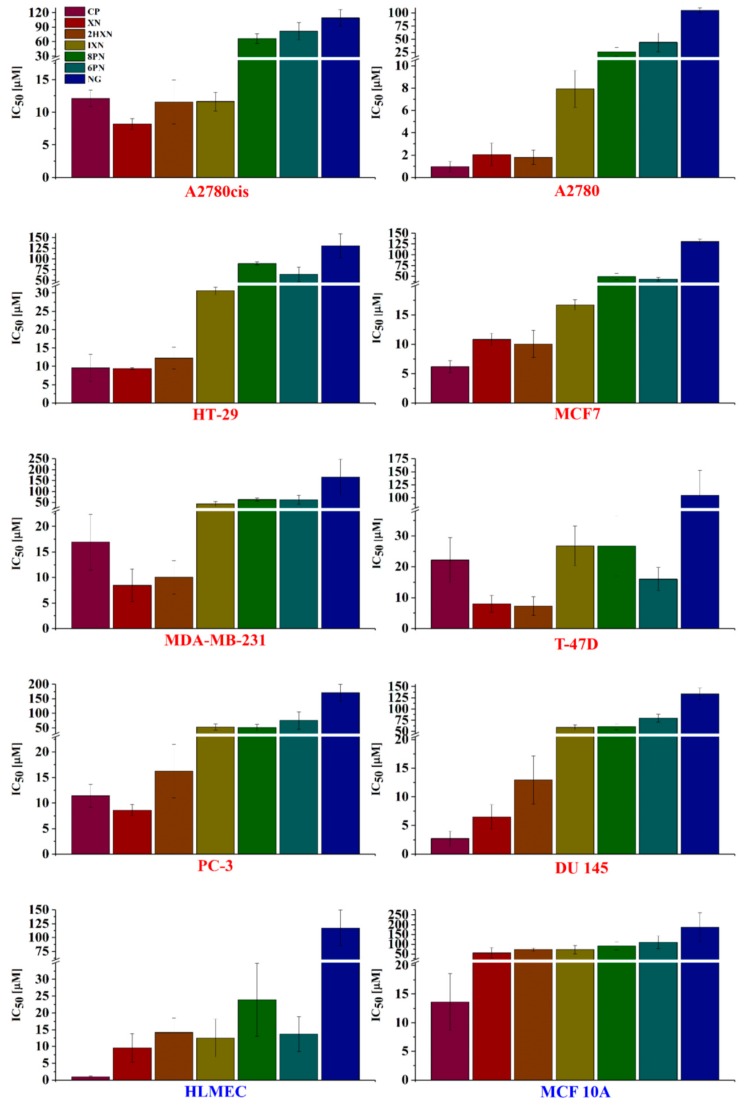
Antiproliferative activity of tested flavonoids and cisplatin against human cancer cell lines (marked red) and normal ones (marked blue).

The substrates we chose included two chalcones: xanthohumol and dihydroxanthohumol (which do not undergo isomerization to flavanone) and four flavanones: isoxanthohumol (which is considered to be a proestrogen), 8-prenylnaringenin, 6-prenylnaringenin and nonprenylated naringenin, as a model compound. We determined the effect of various structural factors in the substrates on the antiproliferative activity against those selected to the study cell lines: the location and/or the presence of the prenyl group, the chalcone/flavanone skeleton and the presence of an O-methyl group at C-5 in flavanones. Flavonoids presented in Figure 1 were tested for the antiproliferative activity towards eight cancer cell lines including estrogen-dependent (MCF7 and T-47D) and two normal ones (HLMEC and MCF 10A). The in vitro antiproliferative activity was measured using SRB assay. The results are presented in Figure 2 (and in the supplement in Table S1). The antiproliferative activity was measured separately for each of the experiments and given as the average IC_50_ (the concentration of tested compound which inhibits 50% of the cells population). Cisplatin (CP), a common anticancer drug, was used as a positive control.

In the case of MCF7 human breast cancer cell line, the lowest IC_50_ was observed for α,β-dihydroxanthohumol and xanthohumol. It is worth noting that for α,β-dihydroxanthohumol, xanthohumol and 6-prenylnaringenin we observed higher antiproliferative activity against the T-47D cell line than for cisplatin. The position of the prenyl group was very important in this case, because 8-prenylnaringenin exerted a much weaker antiproliferative effect than 6-prenylnaringenin. For the other breast cancer cell lines, such an influence was not observed. Higher antiproliferative activity against all tested breast cancer cell lines observed for chalcones XN and 2HXN, compared to prenylated flavanone 8PN and 6PN, indicates that activity towards these particular cell lines highly depends on the flavonoid skeleton. Among the mammary gland tumor cell lines the most sensitive to the antiproliferative properties of tested compounds were T-47D cells (IC_50_ = 7.99–104.53 µM). This is probably related to the highest level of the estrogen receptor expression in the cells of this line, compared to the rest of the mammary gland tumor cell lines. The least sensitive is MDA-MB-231 cell line (IC_50_ = 8.46–166.09 µM). These cells have a very low level of the estrogen receptor expression or have no estrogen receptor at all.

The highest antiproliferative activity towards ovarian cancer cells was observed also for the chalcones XN and 2HXN. The cells of the cisplatin-resistant A2780cis line were more sensitive to XN than to the reference cisplatin, and the antiproliferative activity of 2HXN and IXN were comparable to cisplatin. Xanthohumol showed a higher antiproliferative effect against PC-3 prostate cancer cell line than cisplatin and the most effectively inhibited growth of the DU 145 prostate cancer cell line.

The highest antiproliferative activity toward the HT-29 colon cancer cell line was observed for xanthohumol (comparable to the reference CP) and also for its saturated derivative α,β-dihydroxanthohumol. Kretzschmar et al. described that flavonoid prenylation significantly increases their estrogenic activity. Changing the position of the isopentenyl group from C-8 to C-6 diminishes this activity [24]. In our studies, the same correlation was observed for the majority of cancer cell lines tested. 8-Prenylnaringenin had a stronger anti-proliferative activity against A2780, A2780cis, PC-3 and DU 145 than its 6PN isomer. However, the opposite tendency was observed for HT-29 and T-47D, where 6-prenylnaringenin was stronger than 8-prenylnaringenin.

Naringenin (**6**) showed the lowest antiproliferative activity among the tested compounds (IC_50_ ranging from 100.05 ± 4.77 [µM] for A-2780 to 187.10 ± 72.41 [µM] for MCF7), but its selectivity towards cancer cell lines compared to two normal cell lines (with the exception of PC-3 and MDA-MB-231) was within the range of selectivity index SI = 0.88–1.87. 

The high anti-proliferative activity of the test compounds against normal vascular endothelial cells may indicate their potential anti-angiogenic activity. Xanthohumol itself is known for its anti-angiogenic activity, described, among others, by Saito et al. [31]. Although the benefits of such anticancer (anti-angiogenic) drugs are widely known, their use is associated with a number of side effects related to various vascular disorders and even vascular regression in normal organs [32].

We investigated how the absence of this double bond along with the presence of the prenyl group affected the activity. Additionally, we used as many as eight cancer cell lines. For A2780, T-47D and MCF7 cell lines α,β-dihydroxanthohumol showed a comparable antiproliferative effect to xanthohumol, whereas for the rest of the tested cells it was less active. Especially in the case of PC-3 and DU 145 cell lines, the IC_50_ value for XN is two-fold lower than for 2HXN. This is probably dependent on the specificity of a cell line.

Isomerisation of the chalcones reduced their antiproliferative activity. Only in the case of the A2780cis ovarian cancer cell line, the IC_50_ value for isoxanthohumol was comparable to α,β-dihydroxanthohumol and slightly lower than for cisplatin.

Structural determinants of polyphenols may inhibit the activity of CYP1A1 and CYP1B1 enzymes related to specific types of cancer, such as lung or colorectal ones. The important factors proved to be the prenyl and the methoxy group at C-6′ in xanthohumol, but the strongest CYP1A2 inhibitor appeared to be isoxanthohumol [21].

By comparing the antiproliferative activity of 8-prenylnaringenin and isoxanthohumol we were able to evaluate the importance of the O-methyl group at C5. For most of the tested cancer cell lines (MCF7, MDA-MB-231, HT-29, A2780, and A2780cis) isoxanthohumol was more active than 8-prenylonaringenin. For the rest of the cell lines the cytotoxic activity of both compounds was similar. 

Comparing the sensitivity of all investigated cell lines to tested flavonoids one can observe that except for the hydrogenation of the α,β-double bond in xanthohumol, most of the structural modifications, such as isomerization of XN to IXN or demethylation of IXN to 8PN led to reduction of the cytostatic activity. Among the tested compounds, cisplatin exhibited higher antiproliferative activity only against three of the examined cancer cell lines: MCF7, A2780 and DU 145, and against one of the lines (HT-29), the activity of cisplatin was comparable to xanthohumol. However, this reference compound showed very high antiproliferative against normal human lung microvascular endothelial cells (HLMEC) and normal breast epithelial cell line (MCF 10A), which was 143 and 126-fold higher, respectively, than for tested flavonoids.

Commonly used anticancer drugs, such as cisplatin or doxorubicin have a big disadvantage of being toxic to both cancer and normal cells. Hence, to solve this problem, there is a search for new effective and safe pharmaceuticals. The selective action of a drug can be expressed by the selectivity index (SI), which is determined by comparing the cytotoxic activity of each compound to each cancerous cell line with that to a normal cell line. SI was calculated as a ratio of IC_50_ for a normal cell line (SI_A_–HLMEC; SI_B_–MCF 10A) to IC_50_ value for the respective cancerous cell line using the following equation:

(1) $$\mathrm{SI}=\frac{{\mathrm{IC}}_{50}\;\mathrm{for}\;\mathrm{normal}\;\mathrm{cell}\;\mathrm{line}}{{\mathrm{IC}}_{50}\;\mathrm{for}\;\mathrm{cancerous}\;\mathrm{cell}\;\mathrm{line}}$$

The SI values higher than 1.0 indicate the compounds of considerable anticancer specificity, and SI much greater than 1.0—highly selective ones.

**Figure 3:**
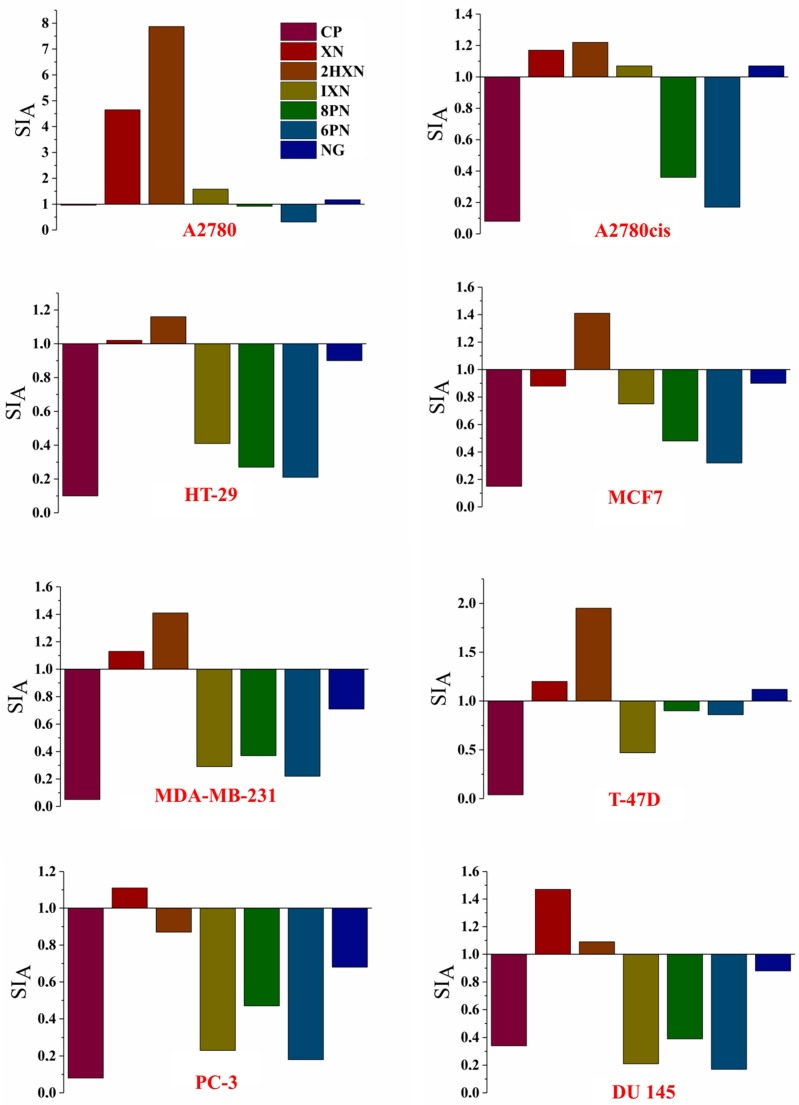
The selectivity indexes (SI_A_) which represents IC_50_ for a normal cell line (HLMEC)/IC_50_ and for a cancerous cell line.

**Figure 4:**
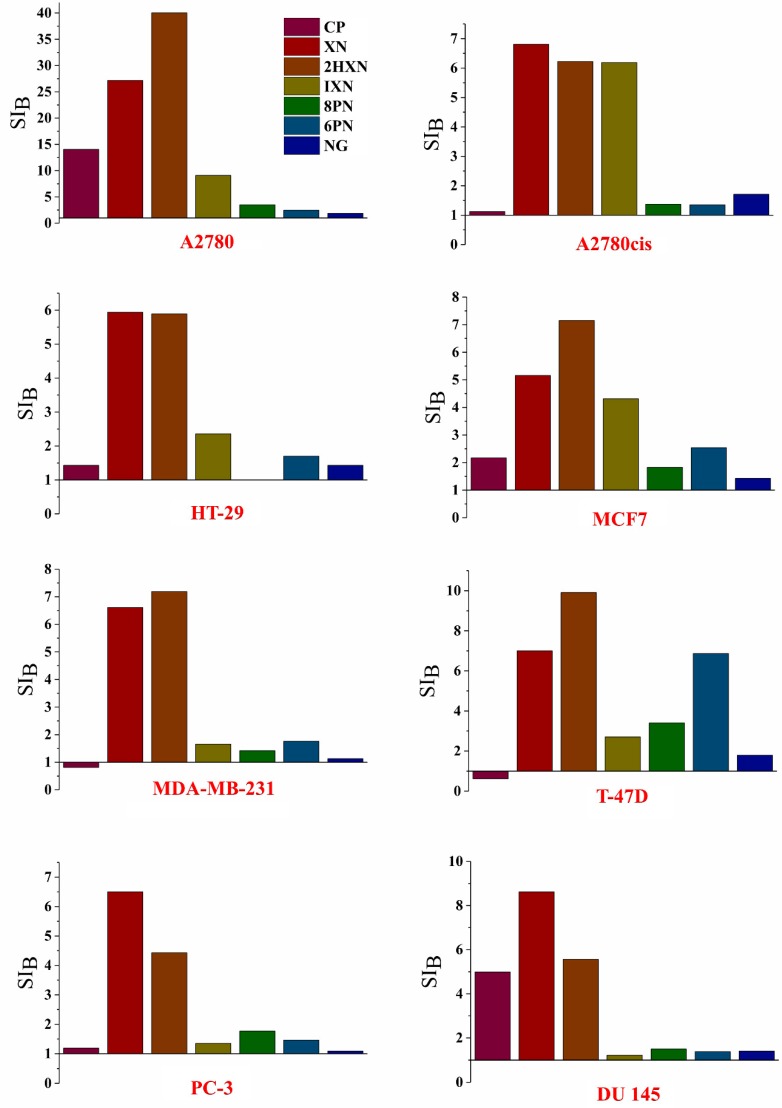
The selectivity indexes (SI_B_) which represents IC_50_ for a normal cell line (MCF 10A) /IC_50_ and for a cancerous cell line.

SI_A_ and SI_B_ calculated for normal cell lines HLMEC and MCF 10A, respectively, are presented in Figure 3 and Figure 4 (for the numerical calculated values see Supplementary Materials).

All investigated compounds were selective towards the MCF 10A cell line (SI_B_ ≥1.00). The chalcones XN and 2HXN exhibited very high selectivity to the breast cancer cell lines (SI_B_ = 5.16–9.91) and the highest one to the ovarian cancer cells (A2780) (SI_B_ 1.94–2.85 times higher than cisplatin). The main prenylflavonoid present in human diet, isoxanthohumol, also showed very high selectivity to the A2780 line, and, what is more important, to cisplatin-resistant ovarian cancer cell line (A2780cis).

The selectivity of the tested compounds towards cancer cell lines in relation to the normal human lung microvascular endothelial cell line is not as high though. The highest SI_A_ value (4.65; 7.87) was noted for the chalcones XN and 2HXN against the A2780 cell line, which is almost 5-8 times higher than the reference. What is the most interesting is xanthohumol and α,β-dihydroxanthohumol were found to be selective agents towards all examined cancer cell lines, with the exception of MCF7 and PC-3 respectively (SI_A_).

The results of our research indicate that all tested flavonoids, with particular emphasis on chalcones, are compounds with selective action, and as dietary supplements, they can effectively prevent carcinogenesis and work in conjunction with a known anticancer drug.

Especially interesting are the results related to the breast cancer cells, because in developed countries they are the most often diagnosed tumors in women along with other estrogen-dependent cancers.

Among the selected in this study of breast cancer and the mammary gland tumor cell lines, the most sensitive to the antiproliferative activity of flavonoids was the T-47D cell line, and the least sensitive were the cells of the MDA-MB-231 line. Our results need further verification in estrogen-sensitive tissue using a proper in vivo model, so as to find whether this activity depends on the estrogen receptor level in the cells.

The examined compounds demonstrated high antiproliferative activity and very high selectivity with respect to cancer cells. They could be used as a dietary supplement, which can effectively prevent carcinogenesis in combination with a known anticancer drug.

## 3. Materials and Methods 

### 3.1. Compounds

The isolation of xanthohumol from spent hops, the isomerization of XN into isoxantohumol, and chemical synthesis of 8-prenylnaringenin were described by Bartmańska et al. [33,34]. α,β-Dihydroxanthohumol was prepared from xanthohumol according to the procedure of the regioselective hydrogenation [35]. Naringenin was purchased from Alexis, Lausen (Switzerland), 6-Prenylnaringenin was obtained by condensation of naringenin with prenyl bromide [36], modified. Chalcones (XN, 2HXN), isoxanthohumol and 8PN had a purity of 99% and 6PN—98%.

The structures of compounds (Figure 1) were confirmed by analysis of NMR spectra, recorded at 600 and 151 MHz on a Bruker Avance 600 DRX spectrometer, 600 MHz (Bruker, Billerica, MA, USA) in DMSO-*d*_6_, CD_3_OD or acetone-*d*_6_. 

Xanthohumol (XN; 3′-(3,3-dimethylallyl)-4,2′,4′-trihydroxy-6′-methoxychalcone], ^1^H NMR (DMSO-*d*_6_) *δ*_H_: 14.65 (1H, s, 2′-OH), 10.61 (1H, s, 4-OH), 10.11 (1H, s, 4′-OH), 7.76 (1H, d, *J* = 15.5 Hz, Hα C=O), 7,65 (1H, d, *J* = 15.5 Hz, Hβ C=O), 7.56 (2H, d, *J* = 8.5 Hz, H-2,6), 6.85 (2H, d, *J* = 8.4 Hz, H-3,5), 6.09 (1H, s, H-5′), 5.14 (1H, t, *J* = 6.8 Hz, H-2”), 3.86 (3H, s, 6′-OCH_3_), 3.14 (2H, d, *J* = 6.9 Hz, H-1”), 1.69 (3H, s, H-4”), 1.60 (3H, s, H-5”). ^13^C NMR *δ*_C_: 191.7 (C=O), 164.7 (C-2′), 162.4 (C-4′), 160.6 (C-6′), 159.9 (C-4), 142.6 (C-β), 130.5 (C-3”), 129.9 (C-2,6), 126.1 (C-1), 123.8 (C-α), 123.0 (C-2”), 116.0 (C-3,5), 107.4 (C-3′), 104.6 (C-1′), 90.1, (C-5′), 55.8 (C-6′-OCH_3_), 25.5 (C-5”), 21.1 (C-1”), 17.7 (C-4”).

α,β-Dihydroxanthohumol (2HXN; 3′-(3,3-dimethylallyl)-4,2′,4′-trihydroxy-6′-methoxy-α,β-dihydrochalcone), ^1^H NMR (CD_3_OD): *δ*_H_ = 7.03 (2H, m, H-2,6), 6.70 (2H, m, H-3,5), 5.98 (1H, s, H-5′), 5.18 (1H, m, H-2”), 3.82 (3H, s, 6′-OCH_3_), 3.20 (2H, m, H-1’’, H-α), 2.83 (2H, m, H-β), 1.75 (3H, s, H-4”), 1.64 (3H, s, H-5”). ^13^C NMR *δ*_C_: 206.1 (C=O), 165.7 (C-2′), 163.7 (C-4′), 162.6 (C-6′), 156.5 (C-4), 133.9 (C-1), 131.3 (C-3”), 130.3 (C-2,6), 124.2 (C-2”), 116.2 (C-3,5), 109.2 (C-3′), 105.8 (C-1′), 91.2 (C-5′), 55.9 (C-6′-OCH_3_), 47.5 (C-α), 31.6 (C-β), 26.0 (C-5”), 22.2 (C-1”), 17.9 (C-4”).

Isoxanthohumol (IXN; 8-(3,3-dimethylallyl)-4,7-dihydroxy-5-methoxyflavanone), ^1^H NMR (acetone-*d*_6_): *δ*_H_ = 10.43 (1H, s, 7-OH), 9.53 (1H, s, 4′-OH), 7.28 (2H, d, *J* = 8.5Hz, H-2′,6′), 6.77 (2H, d, *J* = 8.5 Hz, H-3′,5′), 6.14 (1H, s, H-6), 5.32 (1H, dd, *J* = 12.0, 2.9 Hz, H-2), 5.09 (1H, t, *J* = 7.1 Hz, H-2”), 3.70 (3H, s, 5-OCH_3_), 3.11 (2H, d, *J* = 7.1 Hz, H-1”), 2.92 (1H, dd, *J* = 16.4, 12.4 Hz, H-3ax), 2.57 (1H, dd, *J* = 16.4, 3.0 Hz, H-3eq), 1.58 (3H, s, H-4”), 1.53 (3H, s, H-5”); ^13^C NMR *δ*_C_: 188.8 (C=O), 162.0 (C-5), 161.9 (C-9), 160.1 (C-7), 157.8 (C-4′), 130.5 (C-1′), 130.1 (C-3”), 128.3 (C-2′,6′), 123.3 (C-2”), 115.5 (C-3′,5′), 107.9 (C-8), 105.0 (C-10), 93.1 (C-6), 78.3 (C-2), 55.8 (C-5-OCH_3_), 45.1 (C-3), 26.0 (C-5”), 22.0 (C-1”), 18.0 (C-4”).

8-Prenylnaringenin (8PN; 8-(3,3-dimethylallyl)-5,7,4′-trihydroxyflavanone) ^1^H NMR (DSMO-*d*_6_): δH = 12.10 (1H, s, 5-OH), 10.80 (1H, s, 7-OH), 9.60 (1H, s, 4′-OH), 7.31 (2H, d, *J* = 8.4 Hz, H-2′,6′), 6.78 (2H, d, *J* = 8.6 Hz, H-3′,5′), 5.96 (1H, s, H-6), 5.41 (1H, dd, *J* = 12.7, 3.0 Hz, H-2), 5.08 (1H, t, *J* = 7.3 Hz, H-2″), 3.19 (1H, d, *J* = 7.2 Hz, H-1″), 3.07 (1H, dd, *J* = 17.0, 12.7 Hz, H-3ax), 2.72 (1H, dd, *J* = 17.0, 3.1 Hz, H-3eq), 1.58 (3H, s, H-4″), and 1.53 (3H, s, H-5″). ^13^C NMR: *δ*_C_ = 196.8 (C=O), 164.4 (C-7), 161.2 (C-5), 159.7 (C-9), 157.6 (C-4′), 130.2 (C-3″), 129.3 (C-1′), 128.1 (C-2′,6′), 122.70 (C-2″), 115.2 (C-3′,5′), 107.0 (C-8), 101.8 (C-10), 95.3 (C-6), 78.3 (C-2), 41.9 (C-3), 25.6 (C-5″), 21.3 (C-1″), and 17.6 (C-4″).

6-Prenylnaringenin (6PN; 6-(3,3-dimethylallyl)-5,7,4′-trihydroxyflavanone), ^1^H NMR (acetone-*d*_6_): *δ*_H_ = 12.46 (1H, s, 5-OH), 9.74 (1H, s, 7-OH), 8.64 (1H, s, 4′-OH), 7.40 (2H, d, *J* = 8.2 Hz, H-2′,6′), 6.89 (2H, d, *J* = 8.3 Hz, H-3′,5′), 6.03 (1H, s, H-8), 5.41 (1H, dd, *J* = 13.14, 2.65 Hz, H-2), 5.23 (1H, t, *J* = 7.2 Hz, H-2″), 3.23 (1H, d, *J* = 7.0 Hz, H-1″), 3.15 (1H, dd, *J* = 17.0, 12.9 Hz, H-3ax), 2.72 (1H, dd, *J* = 17.2, 3.1 Hz, H-3eq), 1.75 (3H, s, H-4″), and 1.63 (3H, s, H-5″). ^13^C NMR *δ*_C_ = 196.40 (C=O), 163.7 (C-7), 161.3 (C-5), 161.0 (C-9), 157.7 (C-4′), 130.3 (C-3”), 129.9 (C-1′), 128.2 (C-2′,6′), 122.6 (C-2”), 115.2 (C-3′,5′), 108.0 (C-6), 102.1 (C-10), 94.3 (C-8), 78.9 (C-2), 42.6 (C-3), 24.90 (C-5”), 20.6 (C-1”), and 16.8 (C-4”). 

^1^H NMR and ^13^C NMR spectra of tested compounds can be found in Supplementary Materials.

### 3.2. Cell Lines

The following established in vitro cell lines were applied: MCF7, T-47D, MDA-MB-231 (human breast cancer cell line), A2780 (human ovarian cancer cell line), A2780cis (human cisplatin-resistant ovarian cancer cell line), PC-3 and DU 145 (human prostate cancer cell lines), HT-29 (human colon cancer cell line), HLMEC (human lung microvascular endothelial cell line), and MCF 10A (human mammary epithelial cells line).

The cell lines: MDA-MB-231, HT-29, PC-3, DU 145, and MCF 10A were obtained from the American Type Culture Collection (Rockville, MD, USA), MCF7, T-47D, A2780, A2780cis from the European Collection Cell Cultures (Sigma-Aldrich, Chemie GmbH, Steinheim, Germany), while the HLMEC cell line was obtained by M. Paprocka from the institute [37]. All cell lines are being maintained in the Institute of Immunology and Experimental Therapy (Wrocław, Poland). DU 145 and MCF7 cells were cultured in Eagle medium (supplemented with 4mM L-glutamine, 10% fetal bovine serum, 1 mM sodium pyruvate for DU 145 cells or 2 mM L-glutamine, 1% mem non-essential amino acid solution and 8 µg/mL insulin and 10% fetal bovine serum for MCF7). RPMI 1640 medium was supplemented with 10% fetal bovine serum for A2780 and additionally 1 µM cisplatin for A2780cis. HT-29 and T-47D cell lines were cultured in RPMI 1640 + OPTI-MEM medium supplemented with 2 mM L-glutamine, 5% fetal bovine serum and 8 µg/mL of insulin (T-47D) or 1 mM sodium pyruvate (HT-29). MDA-MB-231, PC-3 and HLMEC cell lines were cultured in RPMI 1640 medium supplemented with 2 mM L-glutamine and 10% fetal bovine serum. Medium for MCF 10A cell line consisted of F-12 nutrient mixture supplemented with cholera toxin (0.05 µg/mL), insulin (10 µg/mL), hydrocortisone (0.5 µg/mL), 5% horse serum, and EGFH (20 ng/mL). All the culture media contained antibiotics: 100 units/mL penicillin (Polfa Tarchomin SA, Warsaw, Poland), and 100 µg/mL streptomycin (Sigma-Aldrich, Chemie GmbH, Steinheim, Germany). All cell lines were grown at 37 °C with 5% CO_2_ humidified atmosphere.

### 3.3. Antiproliferative Assay In Vitro

Twenty-four hours before the incubation with the tested compounds, the cells were seeded in 96-plates (Sarstedt, Germany) at a density of 1 × 104 cells per well (0.75 × 104 per well for MCF7 and 1 × 103 per well for HLMEC) in 100 µL of a culture medium.

Each agent at each concentration (from 0.1 to 100 µg/mL) was tested in a single experiment, which was repeated four to five times. DMSO, which was used as a solvent (in a dilution corresponding to its highest concentration applied to tested compounds), did not exert any inhibitory effect on cell proliferation. Cisplatin was used as a reference drug.

The results were calculated as the IC_50_ (inhibitory concentration 50%)—the concentration of the tested compound which inhibits 50% of the cells population. IC_50_ values were calculated for each experiment separately and mean values ± SD are presented in the Table S1 in the Supplementary Materials. Each compound at each concentration was tested in triplicate in a single experiment, which was repeated four to five times.

#### SRB Assay

An assay was performed after 72 h of exposure to varying concentrations of the tested compounds (from 0.1 to 100 µg/mL); the time of incubation was chosen according to studies of Yong et al. [38] who analyzed the various effects of xanthohumol in 24, 48 and 72 h incubation periods and find out that the majority of xanthohumol-induced effects occurred significantly in the 72 h treatment period. The cells were fixed in situ by gently adding cold 50% trichloroacetic acid (50 µL per well). The plates were incubated at 4 °C for 1h and then washed five times with tap water. Next, the cellular material fixed with TCA was stained by addition to each well 50 µL of 0.4% sulforhodamine B dissolved in 1% acetic acid and incubated for 30 min at room temperature. Unbound dye was removed by washing the plates five times with 1% acetic acid, whereas the protein-bound dye was extracted with 150 µL of 10 mM unbuffered Tris base for determination of the optical density (λ = 540 nm) in Synergy H4 multi-mode microplate reader (Bio Tek Instruments, Winooski, VT, USA).

## 4. Conclusions

Tested flavonoids have antiproliferative activity against human cancer cell lines and they are very selective.

The anticancer activity is related to the structures of flavonoids. Comparing the antiproliferative activity and cancer selectivity of tested compounds, we came to the conclusion that there are chalcones that are promising potential chemotherapeutics, especially to these cancer lines, to which they showed higher antiproliferative activity than cisplatin (IC_50_ [µM]) and their selectivity indexes (SI) were much greater than one, i.e., T-47D, MDA-MB-231, and A2780cis.

The most promising compound concerning high antiproliferative activity against tested human cancer cell lines is xanthohumol, which is inexpensively accessible from waste (spent hops). However, in comparison to the activity on normal cell lines (selective action), it seems that chalcone 2HXN is even more interesting in the context of using it as a potent and selective anticancer drug. As far as the antiproliferative activity is concerned, the presence of a prenyl group is necessary, whereas O-methylation of the hydroxyl group at C-5 increased the cytotoxicity of the compounds against all tested cancer cell lines. The prenyl group at C-8 increased the antiproliferative activity, with the exception of the breast cancer cell lines. For these lines, especially T-47D, it was 6-prenylnaringenin that proved to be more active (IC_50_ lower than for cisplatin) and more selective.

The results of our research provide valuable information, which after the complementary in vivo study may be useful for designing new medicines that are safe and devoid of harmful side effects. 

## Supplementary Materials

Supplementary Materials are available online.
