# Supplementary material for: Highly Cancer Selective Antiproliferative Activity of Natural Prenylated Flavonoids
Source: Molecules. 2018 Nov 9;23(11):2922. doi: 10.3390/molecules23112922 (PMC6278664; doi:10.3390/molecules23112922)

Supplementary data

Antiproliferative activity of hop flavonoids

<u>**Table of Contents:**</u> **Page**

Table S1. ***In vitro*** **antiproliferative activity of tested compounds** **S2**

Table S2. The selectivity indexes SI_A and SI_B of tested compounds **S3**

NMR Spectra **S4-S8**

*^{1}H NMR and ^{13}CNMR spectra of xanthohumol (**XN**)* S4

*^{1}H NMR and ^{13}CNMR spectra spectrum of α,β-dihydroxanthohumol **(2HXN)*** S5

*^{1}H NMR and ^{13}CNMR spectra spectrum of isoxanthohumol **(IXN)*** S6

*^{1}H NMR and ^{13}CNMR spectra spectrum of 8-prenylnaringenin **(8PN)*** S7

*^{1}H NMR and ^{13}CNMR spectra spectrum of 6-prenylnaringenin **(6PN)*** S8

Table S1. *In vitro antiproliferative activity of tested compounds against human cancer cell lines (top) and human normal cell lines (bottom)*

Compound	Cancer cell line, IC_{50} [µM]			
	**MCF7**	**T-47D**	**MDA-MB-231**	**HT-29**
Cisplatin (CP)	6.29 ± 1.05	22.16 ± 7.32	16.93 ± 5.45	9.56 ± 3.75
XN	10.84 ± 0.32	7.99 ± 2.77	8.46 ± 3.19	9.42 ± 0.25
2HXN	10.07 ± 2.31	7.27 ± 3.05	10.02 ± 3.26	12.23 ± 2.99
IXN	16.73 ± 0.88	26.75 ± 6.44	43.34 ± 10.32	30.59 ± 1.00
8PN	49.53 ± 7.36	26.71 ± 9.70	63.81 ± 7.27	89.84 ± 3.42
6PN	43.25 ± 4.37	16.01 ± 3.74	62.64 ± 19.54	64.61 ± 17.07
NG	130.79 ± 6.11	104.53 ±48.31	166.09 ± 82.44	130.80 ± 28.19

Compound	Cancer cell line, IC_{50} [µM]			
	**A-2780**	**A-2780cis**	**PC-3**	**Du-145**
Cisplatin (CP)	0.97 ± 0.46	12.13 ± 1.27	11.43 ± 2.23	2.73 ± 1.32
XN	2.06 ± 1.03	8.21 ± 0.83	8.61 ± 1.11	6.49 ± 2.14
2HXN	1.80 ± 0.64	11.59 ± 3.36	16.27 ± 5.22	12.96 ± 4.20
IXN	7.93 ± 1.65	11.65 ± 1.44	53.24 ± 10.59	59.17 ± 5.73
8PN	25.91 ± 8.32	66.37 ± 10.14	51.36 ± 11.31	60.58 ± 6.66
6PN	44.16 ± 14.71	81.73 ± 17.68	75.53 ± 29.79	79.56 ± 8.89
NG	100.05 ± 4.77	109.23 ± 16.98	171.23 ± 28.78	133.66 ± 12.92

Compound	Normal cell line, IC_{50} [µM]	
	**HLMEC**	**MCF-10A**
Cisplatin (CP)	0.93 ± 0.28	13.63 ± 4.93
XN	9.57 ± 4.23	55.95 ± 27.31
2HXN	14.17 ± 4.24	72.05 ± 8.55
IXN	12.50 ± 5.65	72.12 ± 21.66
8PN	23.91 ± 10.86	90.72 ± 19.80
6PN	13.69 ± 5.16	110.06 ± 32.95
NG	117.24 ± 32.27	187.10 ± 72.41

Table 2. *The selectivity indexes SI_A and SI_B of tested compounds*

Compound	Cancer cell line							
	MCF-7		T-47D		MDA-MB-231		HT-29	
	Selectivity index							
	SI_A	SI_B	SI_A	SI_B	SI_A	SI_B	SI_A	SI_B
Cisplatin (CP)	0.15	2.17	0.04	0.62	0.05	0.81	0.10	1.43
XN	0.88	5.16	1.20	7.00	1.13	6.61	1.02	5.94
2HXN	1.41	7.15	1.95	9.91	1.41	7.19	1.16	5.89
IXN	0.75	4.31	0.47	2.70	0.29	1.66	0.41	2.36
8PN	0.48	1.83	0.90	3.40	0.37	1.42	0.27	1.00
6PN	0.32	2.54	0.86	6.87	0.22	1.76	0.21	1.70
NG	0.90	1.43	1.12	1.79	0.71	1.13	0.90	1.43

Compound	Cancer cell line							
	A-2780		A-2780cis		PC-3		Du-145	
	Selectivity index							
	SI_A	SI_B	SI_A	SI_B	SI_A	SI_B	SI_A	SI_B
Cisplatin (CP)	0.96	14.05	0.08	1.12	0.08	1.19	0.34	4.99
XN	4.65	27.16	1.17	6.81	1.11	6.50	1.47	8.62
2HXN	7.87	40.03	1.22	6.22	0.87	4.43	1.09	5.56
IXN	1.58	9.09	1.07	6.19	0.23	1.35	0.21	1.22
8PN	0.92	3.50	0.36	1.37	0.47	1.77	0.39	1.50
6PN	0.31	2.49	0.17	1.35	0.18	1.46	0.17	1.38
NG	1.17	1.87	1.07	1.71	0.68	1.09	0.88	1.40

Selectivity index (SI) was calculated for each compound using the following formula: $SI_A = IC_{50}$ for normal cell line (HLMEC)/IC_{50} for respective cancerous cell line as indicated on each plot; $SI_B = IC_{50}$ for normal cell line (MCF-10A)/IC_{50} for respective cancerous cell line as indicated on each plot. SI >1.0 indicates a drug with the efficacy against tumor cells greater than the toxicity towards normal cells. SI < 1.0 non-selective action.

Figure S1. *^{1}H NMR and ^{13}CNMR spectra of xanthohumol (XN)*

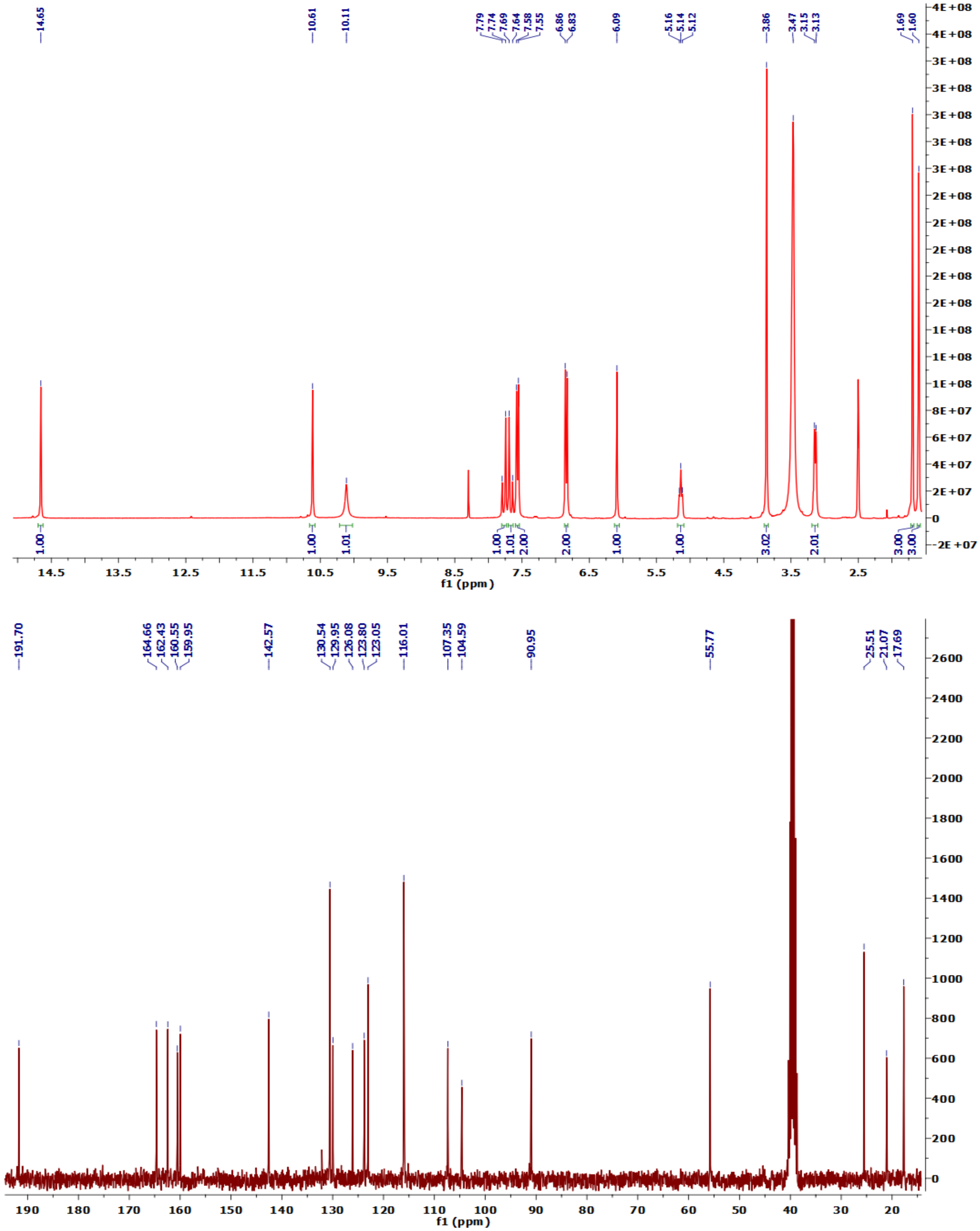

Figure S2. *^{1}H NMR and ^{13}CNMR spectra spectrum of α,β-dihydroxanthohumol (2HXN)*

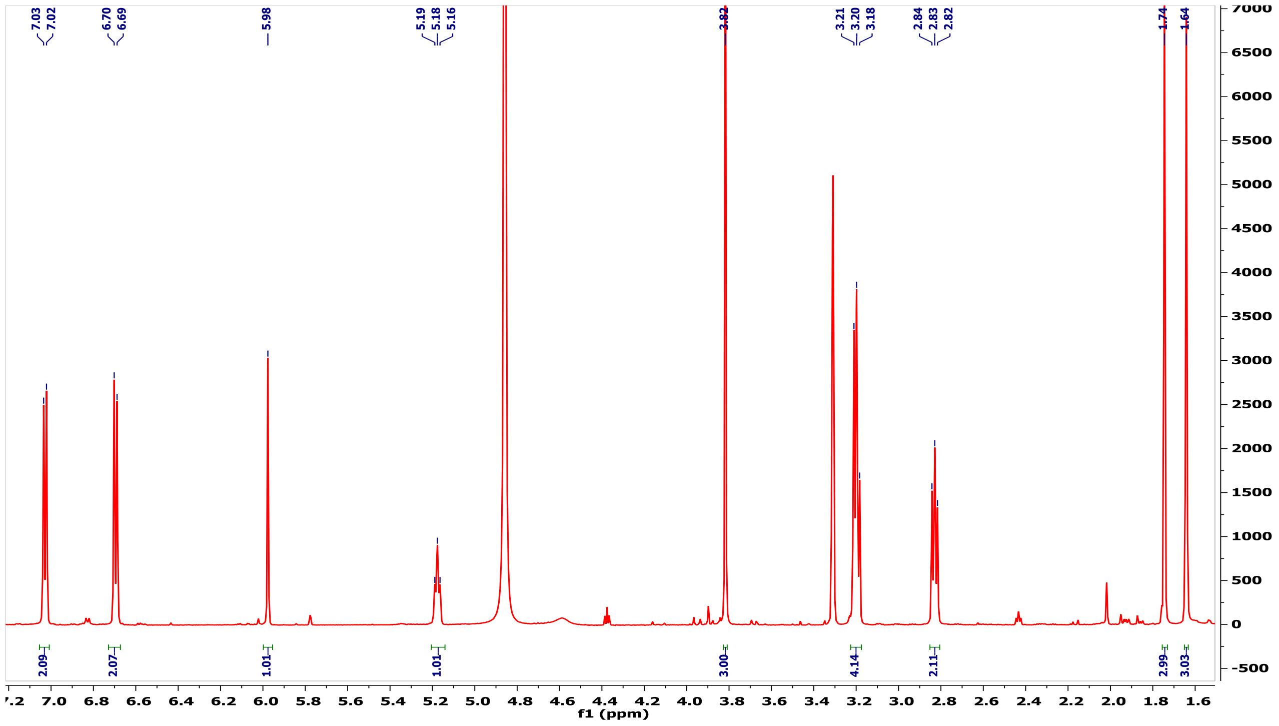

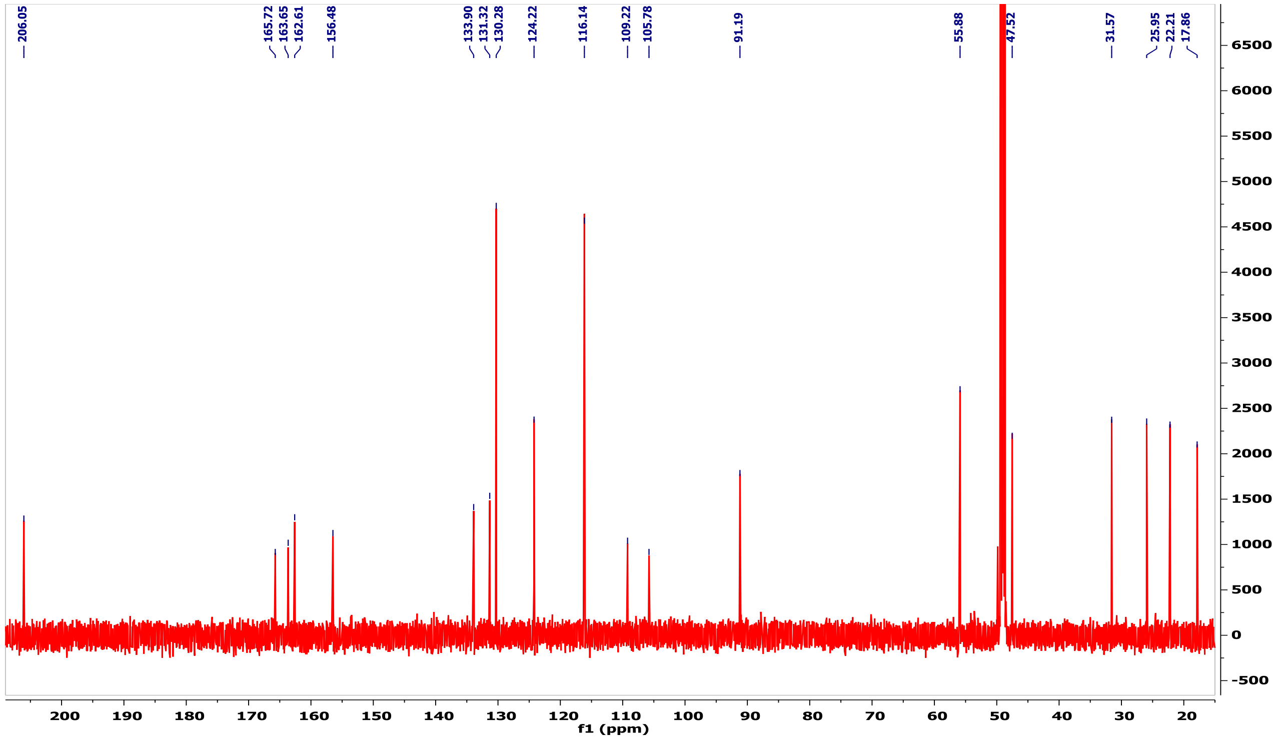

Figure S3. *^{1}H NMR and ^{13}CNMR spectra spectrum of isoxanthohumol (IXN)*

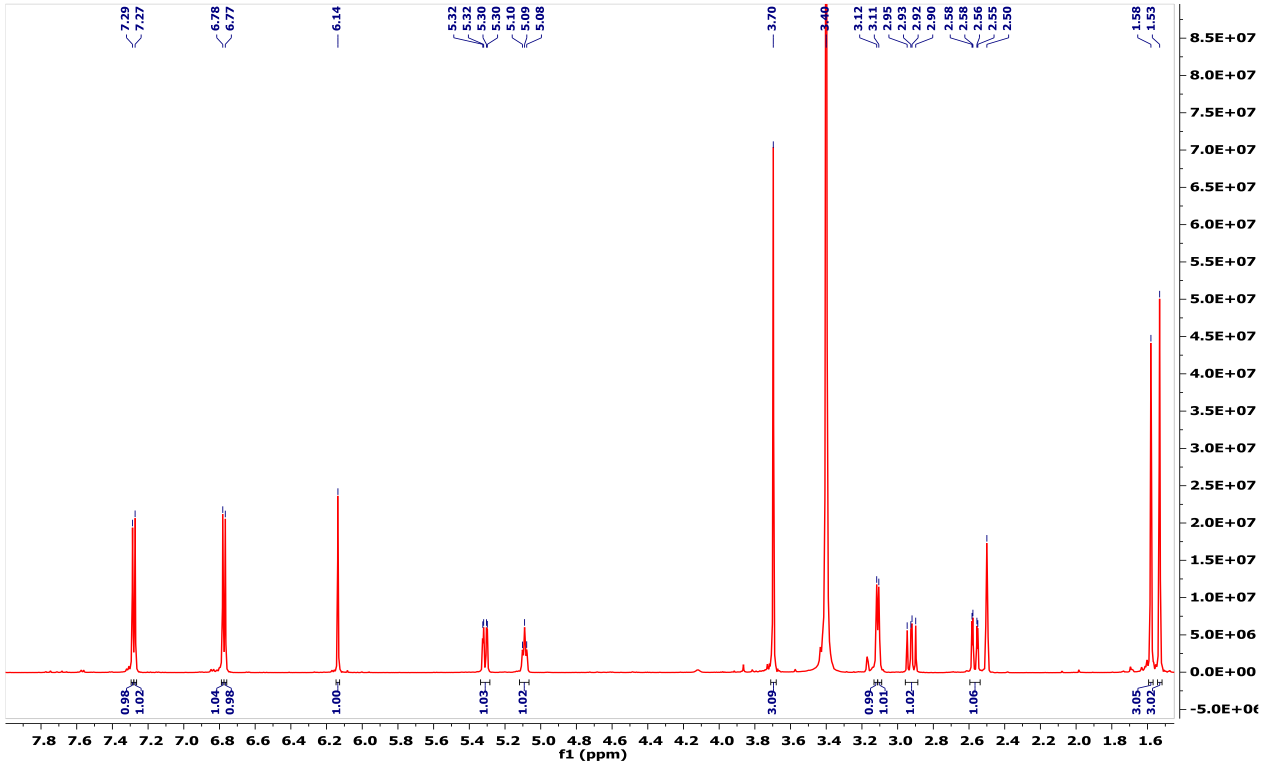

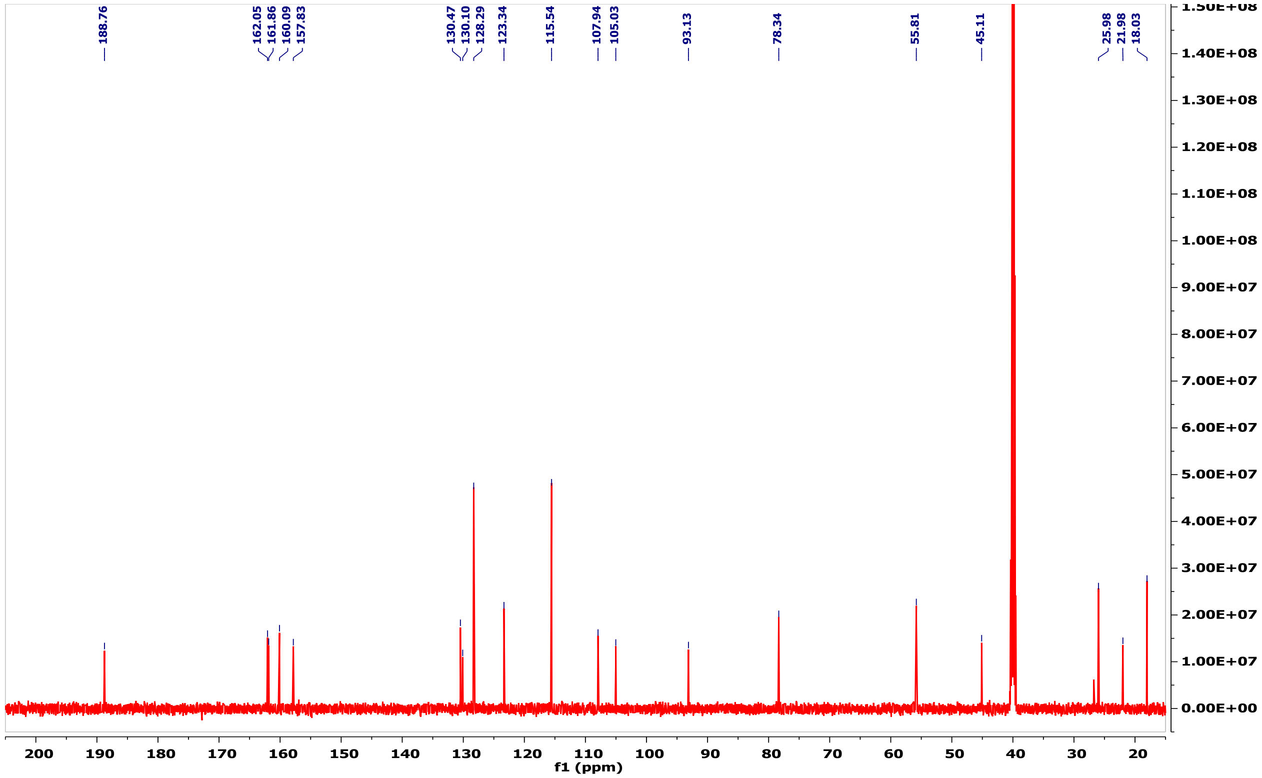

Figure S4. *^{1}H NMR and ^{13}CNMR spectra spectrum of 8-prenylnaringenin (8PN)*

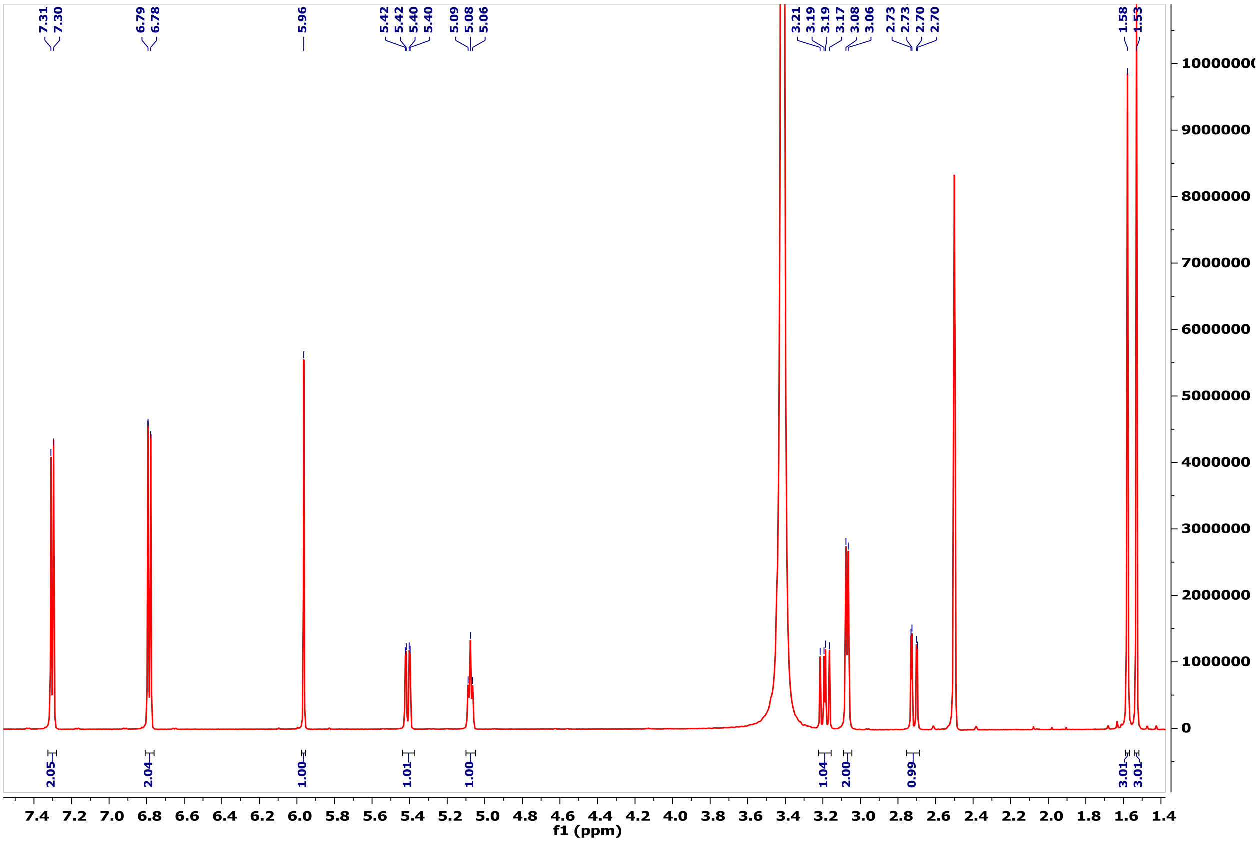

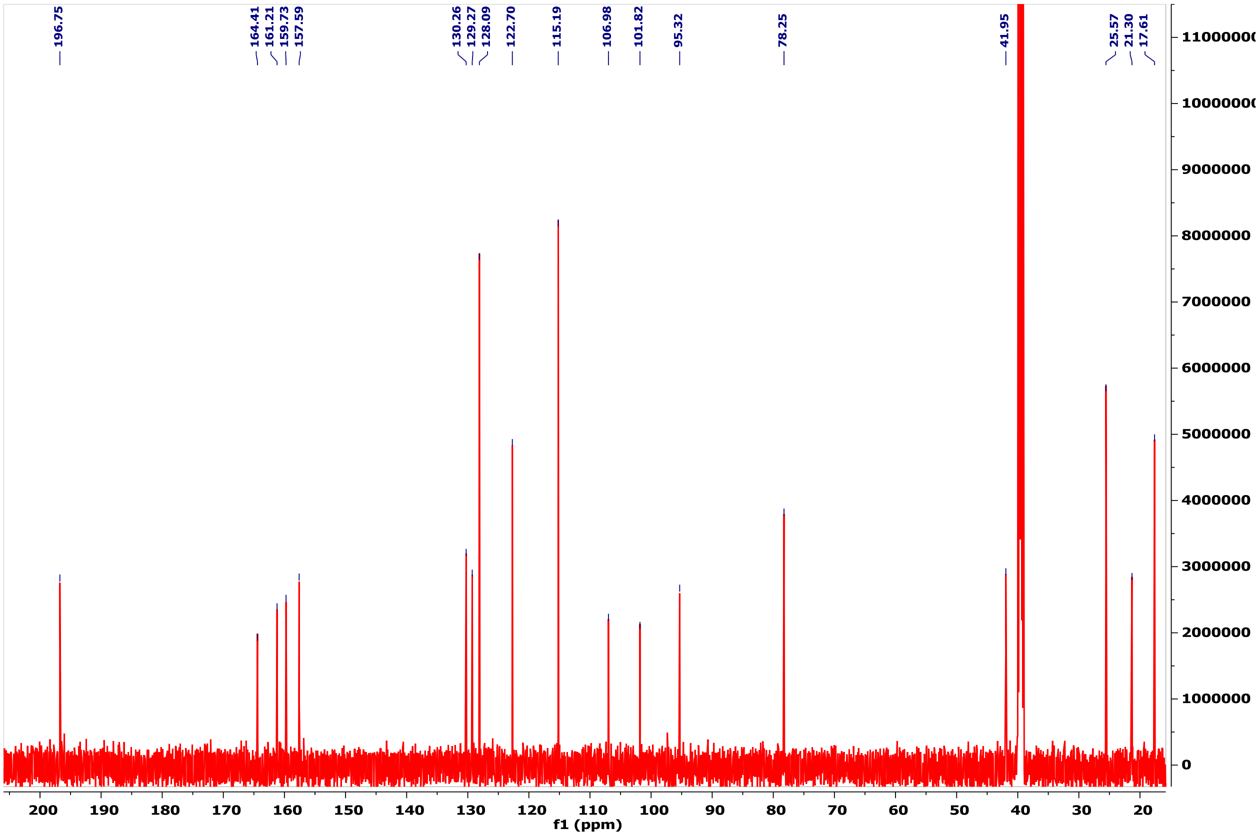

Figure S5. *^{1}H NMR and ^{13}CNMR spectra spectrum of 6-prenylnaringenin (6PN)*

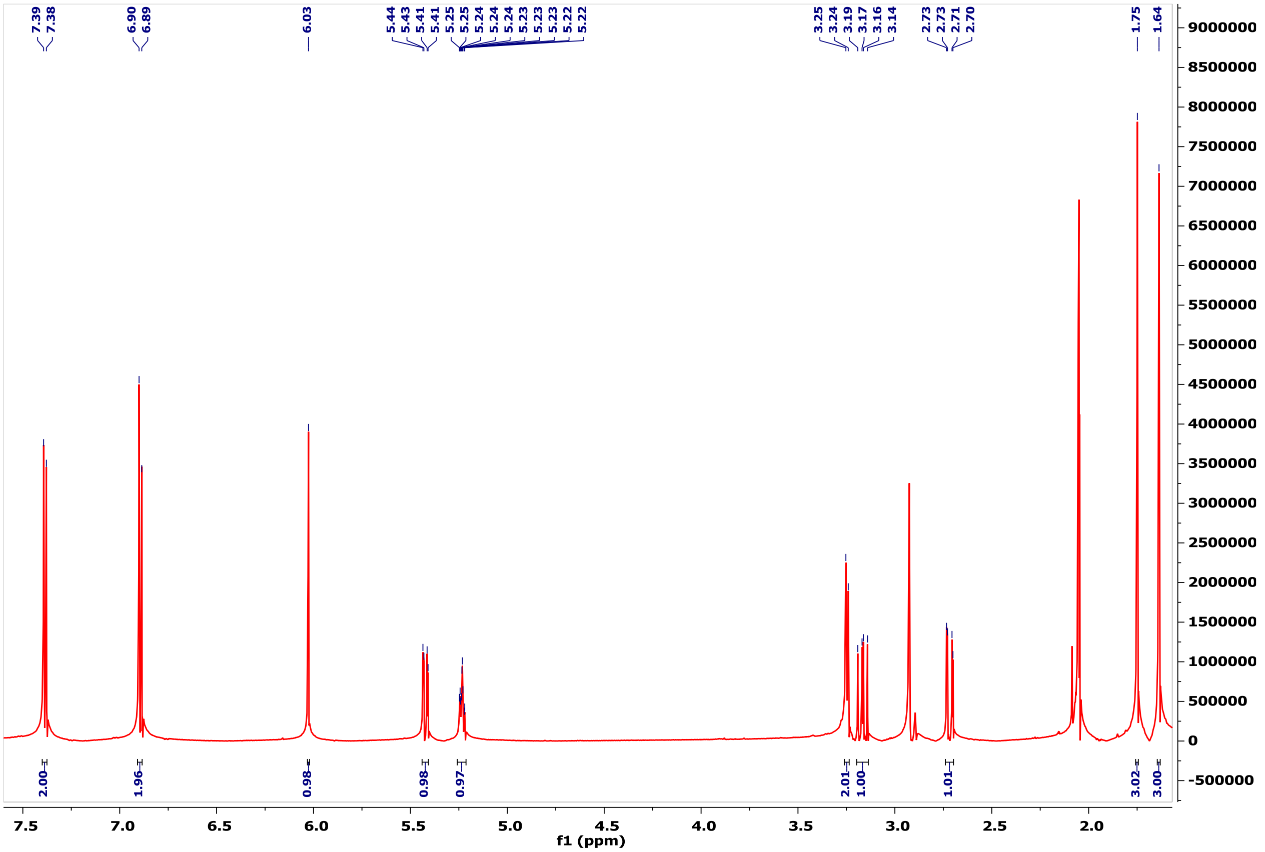

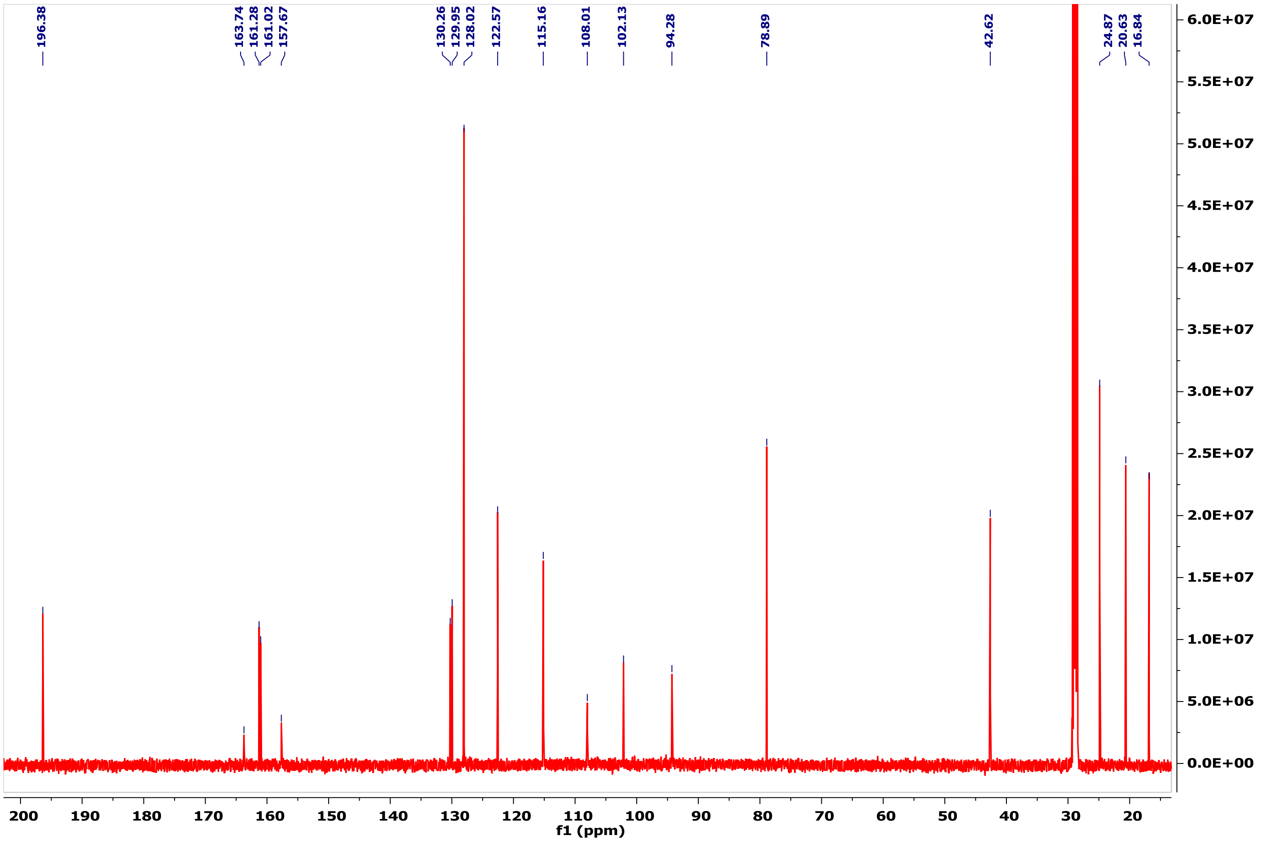

Supplement: Supplementary file 1 [file molecules-23-02922-s001.pdf]
